# A new *Vaejovis* C.L. Koch, 1836, the second known *vorhiesi* group species from the Santa Catalina Mountains of Arizona (Scorpiones, Vaejovidae)

**DOI:** 10.3897/zookeys.270.4500

**Published:** 2013-02-18

**Authors:** Richard F. Ayrey, Michael M. Webber

**Affiliations:** 1 P.O. Box 2236, Flagstaff, Arizona 86003, USA; 2 School of Life Sciences, University of Nevada Las Vegas, 4505 South Maryland Parkway, Las Vegas, Nevada 89154-4004, USA

**Keywords:** Madrean sky islands, speciation, taxonomy, *Vaejovis vorhiesi* group

## Abstract

A new species of the *vorhiesi* group of *Vaejovis* C.L. Koch, 1836, *Vaejovis brysoni* **sp. n.**, is described from the Santa Catalina Mountains in southern Arizona. *Vaejovis deboerae* Ayrey also inhabits this mountain range, making this the first documented case of two *vorhiesi* group species distributed on the same mountain. When compared to all other *vorhiesi* group species, *Vaejovis brysoni* **sp. n.** is distinct based on several combinations of morphological characters and morphometric ratios.

## Introduction

For over 50 years, only four species of montane scorpions in the specious genus *Vaejovis* were known from the topographically complex states of Arizona, New Mexico and Sonora. That number has more than doubled over the past six years, with a total of 13 species now known (see Graham et al. 2012), all belonging to the *Vaejovis vorhiesi* group (Soleglad and Fet 2008). All 13 species have allopatric distributions in Arizona (Sissom et al. 2012), and no records of co-occurrence have been documented. Interestingly, however, several species are distributed across overlapping ecological communities. For example, *Vaejovis jonesi* Stahnke (1940) inhabits rocky juniper woodlands on the Colorado Plateau, and *Vaejovis lapidicola* Stahnke (1940) is distributed across pine-oak woodlands along the southern edge of the Colorado Plateau. These ecological communities overlap across the rim of the Mogollon Plateau, yet to date *Vaejovis jonesi* and *Vaejovis lapidicola* have not been found syntopically. *Vaejovis deboerae* Ayrey (2009) was recently described from the high pine-oak forests of the Santa Catalina Mountains in southern Arizona. The type series was collected at an elevation of 2142 m. Other records suggest that *Vaejovis deboerae* may range as high as 2800 m and as low as 1520 m (Sissom et al. 2012). This vertical distribution encompasses a gradient of ecological communities, ranging from cold pine forest on the high peaks of the Santa Catalina to drier juniper desert scrub in the lower canyons. Recent collecting in the Santa Catalina along the transition zone between desert grassland and pine-oak forest revealed a distinct second species of *Vaejovis vorhiesi* group scorpion. Here we describe this new species, which represents the first record of two *vorhiesi* group species inhabiting the same mountain range.

## Materials and methods

The systematics adhered to in this paper follow the classification established in Fet and Soleglad (2005) and as modified by Soleglad and Fet (2006), Graham and Soleglad (2007), Fet and Soleglad (2007), Soleglad et al. (2007), and Soleglad and Fet (2008).Measurements are as described in Stahnke (1970), trichobothrial patterns are as in Vachon (1974), and pedipalp finger dentition follows Soleglad and Sissom (2001).

Acronyms of depositories — RFA, Richard F. Ayrey; MES, Michael E. Soleglad; USNM, United States National Museum, Smithsonian.

*Material* –In addition to the type material listed below for the new species, the following additional specimens were examined:

*Vaejovis brysoni* sp. n. USA: Arizona: Pima Co.: above Molino Basin on Catalina Highway near Seven Cataracts Vista, Santa Catalina Mountains. 32.35796°N, 110.72538°W, 1626 m. 16 March 2012. R.W. Bryson Jr. 1 ♂, 7 ♀ (RFA). Same locality. 5 April 2012. R.W. Bryson Jr. & D. Hartman 8 ♀ (RFA). Same locality. 18 August 2012. R. F. Ayrey & M. DeBoer-Ayrey. 8 ♀ (RFA).

*Vaejovis cashi* Graham, 2007. USA: Arizona: Cochise Co.: Cave Creek Canyon, Chiricahua Mountains. 2 August 2008. R. F. Ayrey & M. M. DeBoer-Ayrey 4 ♂, 4 ♀ (RFA). Same locality. 23 August 2011. R. F. Ayrey & M. M. DeBoer-Ayrey 3 ♂, 4 ♀ (RFA).

*Vaejovis crumpi* Ayrey et Soleglad, 2011. USA: Arizona: Yavapai Co.: by Lynx Lake, Prescott. 14 August 2008. R. F. Ayrey & M. M. DeBoer-Ayrey 3 ♂, 5 ♀ topotypes (RFA). Same locality. 14 September 2009. R. F. Ayrey & M. M. DeBoer-Ayrey 4 ♂, 4 ♀ (RFA). Same locality. 8 August 2010. R. F. Ayrey & M. M. DeBoer-Ayrey 3 ♂, 5 ♀ (RFA).

*Vaejovis deboerae* Ayrey, 2009. USA: Arizona: Pima Co.: Rose Canyon Campground, Santa Catalina Mountains. 28 August 2011. R. F. Ayrey & M. M. DeBoer-Ayrey 3 ♂, 5 ♀ (RFA). Same locality. 29 August 2011. R. F. Ayrey & M. M. DeBoer-Ayrey 4 ♂, 4 ♀ (RFA).

*Vaejovis electrum* Hughes, 2011. USA: Arizona: Graham Co.: Upper Arcadia Campground, Mount Graham. 17 July 2009. R. F. Ayrey & M. M. DeBoer-Ayrey 2 ♂, 6 ♀ (RFA). USA: Arizona: Graham Co.: 9415 feet asl, Mt Graham Hwy., Mt. Graham. 18 July 2009. R. F. Ayrey & M. M. DeBoer-Ayrey 1 ♂, 4 ♀ (RFA).

*Vaejovis feti* Graham, 2007. USA: New Mexico: Meadow Creek, Black Mountains. 6 July 1978. M. H. Muma 4 ♂, 3 ♀ (MES).

*Vaejovis halli* Ayrey, 2012. USA: Arizona: Gila Co.: Mount Ord. 11 September 2010. R. F. Ayrey & M. M. DeBoer-Ayrey 2 ♂, 6 ♀, paratypes (RFA). Same locality. 2 May 2011. R. F. Ayrey & M. M. DeBoer-Ayrey 3 ♂, 5 ♀, paratypes (RFA).

*Vaejovis jonesi* Stahnke, 1940. USA: Arizona: Coconino County: near Wupatki National Monument. 1 April 2011. R. F. Ayrey. 1 ♀ topotype (RFA).

*Vaejovis lapidicola* Stahnke, 1940. USA: Arizona: Coconino County: Red Sandstone Quarry, Flagstaff. 1 June 2011. R. F. Ayrey & M. M. DeBoer-Ayrey 1 ♂, 7 ♀ topotypes (RFA).

*Vaejovis paysonensis* Soleglad, 1973. USA: Arizona: Coconino County: Control Road, 25 miles East of Payson. 5 July 2011. R. F. Ayrey & M. M. DeBoer-Ayrey 1 ♂, 7 ♀ topotypes (RFA). Same locality. 6 July 2011. R. F. Ayrey & M. M. DeBoer-Ayrey 2 ♂, 6 ♀ topotypes (RFA).

*Vaejovis tenuipalpus* Sissom et al., 2012. USA: Arizona: Mojave Co.: Getz Peak, Hualapai Mountains. 9 August 2009. R. F. Ayrey & M. M. DeBoer-Ayrey 1 ♂, 7 ♀ paratopotypes (RFA).

*Vaejovis vorhiesi* Stahnke, 1940. USA: Arizona: Cochise Co.: Miller Canyon, Huachuca Mountains. 24 May 2011. R. F. Ayrey & M. M. DeBoer-Ayrey 1 ♂, 7 ♀ topotypes (RFA). Garden Canyon, Huachuca Mountains. 26 August 2011. R. F. Ayrey & M. M. DeBoer-Ayrey 4 ♂, 6 ♀ (RFA). Lutz Canyon, Huachuca Mountains. 27 March 2011. R. F. Ayrey & M. M. DeBoer-Ayrey 2 ♂, 2 ♀ (RFA).

## Taxonomy

### Order Scorpiones C. L. Koch, 1850

Suborder Neoscorpiones Thorell et Lindström, 1885

Infraorder Orthosterni Pocock, 1911

Parvorder Iurida Soleglad et Fet, 2003

Superfamily Chactoidea Pocock, 1893

Family Vaejovidae Thorell, 1876

Subfamily Vaejovinae Thorell, 1876

#### Vaejovis brysoni sp. n.

urn:lsid:zoobank.org:act:80FC6074-1CD9-4DED-B155-2F7FE348495C

http://species-id.net/wiki/Vaejovis_brysoni

**Figure 1.**
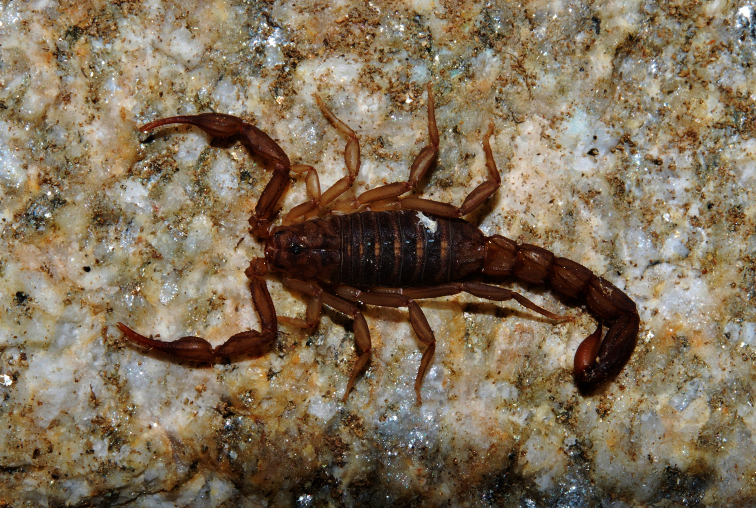
*Vaejovis brysoni*sp. n., paratype female in natural habitat.

Figs 1 [Fig F2] [Fig F3] [Fig F4] –10 12 Table 1

##### Type material.

Female holotype.USA: Arizona: Pima Co.: above Molino Basin on Catalina Highway near Seven Cataracts Vista, Santa Catalina Mountains. 32.35796°N, 110.72538°W, 1626 m. 16 March 2012. R.W. Bryson Jr. (RFA specimen number 632, deposited in USNM). Paratypes. Same locality as holotype. 16 March 2012. R.W. Bryson Jr. 1 ♂ (RFA specimen number 633) 2 ♀ (RFA specimen numbers 634 and 635). 17 August 2012. R. F. Ayrey. 1 ♀ (RFA specimen number 643).

##### Etymology.

The specific epithet is a patronym honoring our colleague Dr. Robert W. Bryson, Jr., the collector of the holotype.

##### Diagnosis.

Relatively small-bodied scorpion from the Seven Cataracts Overlook area of the Santa Catalina Mountains, southern Arizona (total body length of the female holotype is 27.50 mm). Color is light to medium brown, light brown to yellow on the legs, with underlying dark mottling on carapace and mesosoma. Metasoma is light brown with darker carinae.

Significant characters that distinguish *Vaejovis brysoni* sp. n. from other known species in the *vorhiesi* group are described below.

*Vaejovis jonesi*, *Vaejovis lapidicola*, *Vaejovis paysonensis*, *Vaejovis crumpi*, and *Vaejovis bigelowi* all possess 7 inner denticles (*ID*) on the chela movable finger, not 6 as in *Vaejovis brysoni* sp. n. The new species can be distinguished from *Vaejovis halli* by having significantly larger metasomal segment L/W ratios on I, II, and V (Table 1). *Vaejovis brysoni* can be distinguished from *Vaejovis bandido* by having larger metasomal segment I L/W ratios in addition to larger fixed finger L/chela L ratios. *Vaejovis brysoni* sp. n. can be distinguished from *Vaejovis deboerae* by having a smaller and less-developed subaculear tubercle. *Vaejovis brysoni* sp. n. also have shorter total body lengths and shorter carapace lengths. In addition, *Vaejovis deboerae* have larger telson vesicle L/W ratios. However, *Vaejovis brysoni* sp. n. have larger metasomal segment I L/W ratios and larger fixed finger L/chela L ratios. *Vaejovis brysoni* sp. n. also have fewer pectinal teeth than *Vaejovis deboerae*. *Vaejovis brysoni* sp. n. can be distinguished from *Vaejovis vorhiesi* by having larger metasomal segments L/W ratios on I, II, and III. However, *Vaejovis vorhiesi* have larger chela L/W ratios. *Vaejovis brysoni* sp. n. also have fewer pectinal teeth than *Vaejovis vorhiesi*. *Vaejovis brysoni* sp. n. can be distinguished from *Vaejovis cashi* by having longer total body lengths, and larger metasomal segment L/W ratios on segments I, II, and III. In addition, *Vaejovis brysoni* sp. n. exhibit larger fixed finger L/chela L ratios.*Vaejovis brysoni* sp. n. can be distinguished from *Vaejovis feti* by having larger metasomal segment I and II L/W ratios. Additionally, *Vaejovis brysoni* sp. n. also have larger fixed finger L/chela L ratios than *Vaejovis feti*. *Vaejovis brysoni* sp. n. can also be distinguished from *Vaejovis feti* by having a higher number of pectinal teeth. *Vaejovis brysoni* sp. n. can be distinguished from *Vaejovis electrum* by having larger metasomal segment L/W ratios on segments I, II, III, and V. In addition, *Vaejovis brysoni* sp. n. also have larger fixed finger L/chela L ratios than *Vaejovis electrum*. Finally, *Vaejovis brysoni* sp. n. can be distinguished from *Vaejovis tenuipalpus* by having smaller metasomal segment L/W ratios on segments II, III, and IV. *Vaejovis tenuipalpus* also have larger femur, patella, and chela L/W ratios.

##### Description of the holotype.

Color of the holotype is light to medium brown, light brown to yellow on the legs, with underlying dark mottling on carapace and mesosoma. Metasoma is light brown with darker carinae. Metasomal segments are slightly wider than the vesicle. Small spinoid subaculear tubercle is present (Fig. 1). The pedipalp fixed finger has 5 to 6 ID denticles and movable finger has 6 ID denticles. Carapace:Anterior margin of the carapace is slightly emarginated, the posterior margin is straight. The carapace is moderately granular, with three lateral eyes present on each side. The median furrow is moderate and traverses the entire length of the carapace. The ratio of the location of the median eyes on the carapace (anterior edge/carapace length 0.73/3.75) = 0.19; carapace length/width at median eyes 3.75/2.35 = 1.60. The carapace is longer than metasomal segmentV.Mesosoma: Tergites are moderately granular with vestigial median carina on tergites I–VI. Tergite VII with weak median carina on anterior third and strong dorsal lateral and lateral supramedian granular carinae. Sternites I–V are finely granular and without carinae. Sternite V with weak granular ventral lateral carinae on middle 1/3. Presternites are smooth. Spiracles are ovoid with median side rotated 35 degrees from posterior sternite margin. Sternites with variable number of microsetae. Pectines:Pectinal tooth count is11/12. All pectinal teeth have exterodistal angling with a large sensorial area. Middle lamellae are 6/6. Fulcra are present. Each fulcra with 1–3 central setae.Metasoma: The carapace of the holotype female is longer than the fifth metasomal segment. Ratio of segment I length/width 0.93; of segment II length/width 1.03; of segment III length/width 1.18; of segment IV length/width 1.50; of segment V length/width 2.32. Segments I–IV: dorsolateral carinae are strong and granular to slightly dentate, with the distal denticle of I–IV enlarged and spinoid. Lateral supramedian carinae I–IV are strong and crenulate, with enlarged spinoid distal denticle. Lateral inframedian carinae are moderately granular on posterior 4/5 of segment I, 4/5 of II, 1/2 of III, and nearly obsolete on segment IV. Ventrolateral carinae on segment I, II, and III are moderate and granular; on IV moderate, granular and slightly serrate. Ventral submedian carinae are weak on segment I, weak to moderate on II, moderate, granular to slightly serrate on III and IV. The dorsal and lateral intercarinal spaces are very finely granular. Segment I–IV: ventral submedian setae count is 3/3. Segment V: dorsolateral carinae are moderate and slightly serrate on anterior 1/3. Lateromedian carinae are weak to moderate and granular on basal 3/5, and obsolete on distal 2/5. Ventrolateral and ventromedian carinae are strong and crenate to serrate. Intercarinal spaces are finely granular. Ventrolateral setae count 4/4. Telson:Smooth with four pairs of large setae on the ventral surface, three large setae are along both lateral edges of the vesicle with numerous smaller setae. A small spinoid subaculear tubercle is present. Chelicerae: The dorsal edge of movable cheliceral finger with two subdistal (sd) denticles. Ventral edge is smooth, with well developed serrula on distal half. Pedipalps: Trichobothrial pattern type C (Vachon 1974) (Fig. 12). Trichobothria *ib* and *it* near base of fixed finger. Pedipalp ratios: chela length/width 4.00; femur length/width 2.69; patella length/width 2.59; fixed finger length/carapace length 0.68. Chela:Carinae are moderate. Fixed and movable finger median denticles (MD) are aligned and divided into 6 subrows by 5 outer denticles (OD) and usually 6 ID denticles. Femur: Dorsal internal and external are moderate and granular; ventral internal granular to crenulate; ventral external are slightly serrate; dorsal and ventral surfaces are covered with fine granules; external surface is smooth. Patella:Internal surface are covered with very strong dentate to serrate granules on the *DPSc* carina. Dorsal external and internal are moderate and granular. Ventral internal carinae are strong and granular. External surface is rounded with scattered granulation; dorsal and ventral surfaces are covered with minute granules. Legs: Ventral surface of tarsomere II with single median row of spinules terminating distally with one spinule pair.

##### Variability.

Variability of fixed finger ID denticle count was found. For *Vaejovis brysoni* sp. n., fixed finger ID denticle counts ranged from 5 (n=3) to 6 (n=5). Variation also existed for female *Vaejovis brysoni* sp. n. in the number of pectinal teeth 11/11 (n=2), 11/12 (n=3), 12/11 (n=1), 12/12 (n=2) with a mean of 11.5 for females, and 13/14 for the paratype male (n=1). In addition, there was variation in the number of middle lamellae 5/5 (n=1), 6/6 (n=5), 7/6 (n=1), 7/7 (n=1) and for the paratype male 8/9 (n=1). The right hemispermatophore was extracted from the paratype male. The right hemispermatophore is 3.10 mm in total length, and its lamina is 1.20 mm in length and 0.39 mm in width. The hemispermatophore is lightly sclerotized near the dorsal trough, and possesses a subtle distal crest on the inner distal aspect of the lamina. The lamellar hook is strong and widely bifurcated, and emanates from the dorsal trough. A medium, defined truncal flexure is visible on the external aspect of the trunk/lamina juncture. The male paratype also posseses an area of reduced pigmentation (white patch) on the posterior ¼ of the third sternal plate. (Graham and Bryson 2010).

##### Mensuration

(mm).Female holotype: total length 27.5; carapace length 3.75; mesosoma length 8.13; metasoma length 15.63. Metasoma: segment I length/width/depth 1.81/1.94/1.38; segment II length/width/depth 2.00/1.94/1.25; segment III length/width/depth 2.06/1.75/1.31; segment IV length/width/depth 2.63/1.75/1.25; segment V length/width/depth 3.63/1.56/1.25. Telson: length 3.50; vesicle length/width/depth 2.25/1.25/1.06; aculeus length 1.25. Pedipalps: total length 10.44; femur length/width 2.69/1.00; patella length/width 2.75/1.06; chela length 5.00; palm length/width/depth 2.44/1.25/1.13; movable finger length 2.69; fixed finger length 2.56. Male paratype: total length 21.1; carapace length 2.75; mesosoma length 5.25; metasoma length 13.1; Metasoma: segment I length/width/depth 1.44/1.81/1.25; segment II length/width/depth 1.56/1.88/1.13; segment III length/width/depth 1.75/1.63/1.19; segment IV length/width/depth 2.38/1.63/1.19; segment V length/width/depth 3.25/2.26/1.13. Telson: length 2.69; vesicle length/width/depth 1.81/1.00/1.00; aculeus length 0.88. Pedipalps: total length 9.5; femur length/width 2.44/0.81; patella length/width 2.63/0.88; chela length 4.44; palm length/width/depth 1.88/1.06/1.06; movable finger length 2.69; fixed finger length 2.56.

##### Distribution and natural history.

*Vaejovis brysoni* sp. n. is known only from the type locality above Molino Basin on the Catalina Highway near the Seven Cataracts Vista, Santa Catalina Mountains, Arizona, USA. The type localities of the 12 described species in the *vorhiesi* group from Arizona and western New Mexico are shown in Figure 13. *Vaejovis brysoni* sp. n. is widely allopatric with *Vaejovis halli*, *Vaejovis vorhiesi*, *Vaejovis cashi*, *Vaejovis feti*, and *Vaejovis electrum* (Fig. 13). *Vaejovis brysoni* sp. n. and *Vaejovis deboerae* both occur within the Santa Catalina Mountains, and their ranges may overlap, perhaps along the mid-elevation pine-oak woodlands between 1800–1900 m.

The type specimens were found at night using a UV flashlight alongside the Catalina Highway. This area lies within open oak woodland and the transition zone from drier desert grassland to pine-oak woodland (Whittaker and Niering 1965). Several *Pseudouroctonus apacheanus* (Gertsch & Soleglad, 1972) and *Centruroides sculpturatus* Ewing, 1928 were also observed. In August of 2012, three captive female *Vaejovis brysoni* were observed with first instar juveniles (Fig. 3). The mean juvenile count was 23.67. The 1^st^ instar orientation on the mother’s back was non-random, as is seen with many other species of *Vaejovis* (Hjelle 1974). They were facing anteriorly with the prosoma down and the metasoma raised over the prosoma of the juvenile immediately posterior to them.

**Table 1.** Morphometrics (mm) of female *Vaejovis brysoni* sp.n. versus other *Vaejovis vorhiesi* group species. Data on pectinal teeth with * from (Hughes 2011) and ** from (Sissom et al. 2012). Bold numbers are those which have no overlap with *Vaejovis brysoni* sp. n.

		***Vaejovis brysoni* Ratio Comparisons**							
***Vaejovis* spp.**	***Vaejovis brysoni* (8)**	***Vaejovis bandido* (5)**	***Vaejovis halli* (3)**	***Vaejovis deboerae* (3)**	***Vaejovis vorhiesi* (3)**	***Vaejovis cashi* (3)**	***Vaejovis feti* (3)**	***Vaejovis electrum* (3)**	***Vaejovis tenuipalpus* (3)**
Total length Carapace length Ca L/MetV L	22.88–27.50 2.88–3.75 0.88–1.03	24.65–27.75 3.12–3.38 0.97–0.98	21.87–23.43 2.90–3.18 0.98–0.99	**29.64–33.14 3.79–4.38** 0.89–0.97	24.62–26.55 3.21–3.39 0.98–1.06	**20.90–22.10** 2.86–3.11 0.96–1.14	22.00–23.46 3.01–3.19 0.96–1.05	24.48–25.38 3.48–3.60 1.02–1.13	**28.38–31.24** 3.52–3.71 0.93–0.98
Segment I length/width	0.93–1.00	**0.73–0.78**	**0.70–0.77**	**0.72–0.79**	**0.68–0.73**	**0.61–0.66**	**0.69–0.73**	**0.65–0.68**	0.90–0.94
Segment II length/width	0.90–1.03	0.88–0.94	**0.79–0.87**	0.98–1.03	0.85–0.92	**0.74–0.80**	**0.69–0.73**	**0.68–0.89**	**1.09–1.18**
Segment III length/width	1.00–1.09	0.96–1.04	0.94–1.00	1.02–1.14	**0.96–0.98**	**0.89–0.92**	0.93–1.13	**0.93–0.98**	**1.29–1.36**
Segment IV length/width	1.34–1.61	1.35–1.44	1.27–1.50	1.48–1.60	1.39–1.62	1.28–1.39	1.35–1.60	1.33–1.46	**1.74–1.83**
Segment V length/width	2.15–2.82	2.02–2.16	**1.79–2.11**	2.10–2.32	2.08–2.22	2.05–2.15	2.07–2.24	**1.81–1.91**	2.49–2.52
Telson VesicleLength/width	1.60–2.06	1.59–1.69	1.68–1.85	**2.22–2.43**	1.63–1.72	1.56–1.71	1.62–1.87	1.45–1.62	1.60–1.77
Femurlength/width	2.87–3.22	2.86–3.07	2.83–3.33	2.74–2.90	2.87–3.22	2.74–3.02	2.89–3.41	2.78–3.19	**3.81–3.83**
Patellalength/width	2.47–3.29	2.83–3.21	2.89–3.63	2.91–3.16	3.12–3.19	2.86–3.03	2.53–2.66	2.86–2.94	**3.72–3.85**
Chela length/width Ff L/Ca L Ff L/Ch L	3.71–4.55 0.68–0.83 0.51–0.56	4.09–4.48 0.65–0.69 **0.48–0.49**	4.33–4.79 0.67–0.77 0.46–0.53	4.17–4.53 0.71–0.77 0.48–0.52	**4.57–5.30** 0.72–0.81 0.47–0.54	3.84–4.52 0.68–0.73 **0.47–0.49**	3.59–3.91 0.69–0.80 **0.48–0.50**	3.74–4.19 0.68–0.69 0.47–0.51	**5.05–5.43** 0.83–0.87 0.50–0.51
Pectinal Teeth	11–12 11.5(16)	11–12 11.1(10)	11–13 11.94(16)	12–13 12.17(6)	12–13 12.87(339)*	10–12 10.98(337)*	**10** 10.00(6)	11–12 11.75(96)*	11–12 11.15(26)**

**Figure 2. F2:**
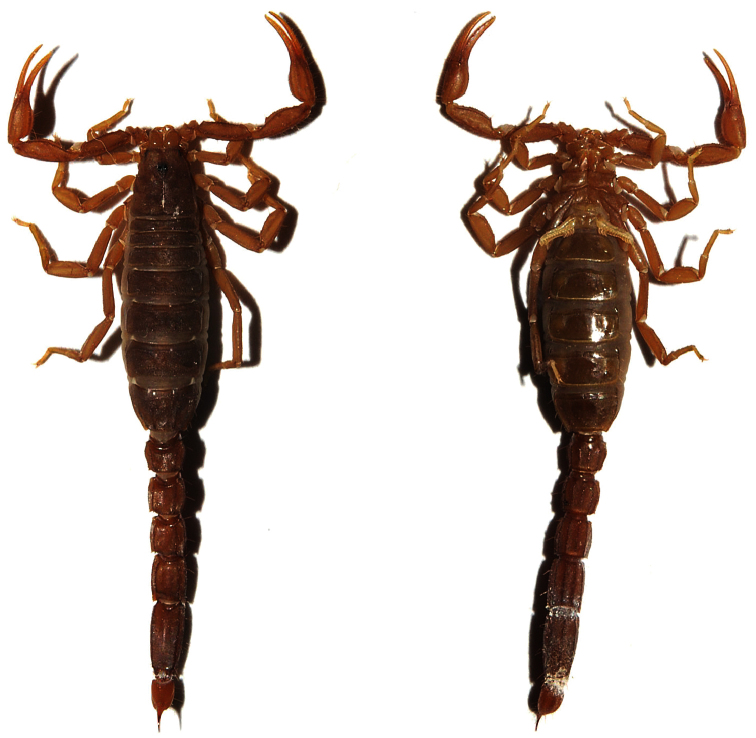
*Vaejovis brysoni* sp. n., paratype female dorsal and ventral views.

**Figure 3. F3:**
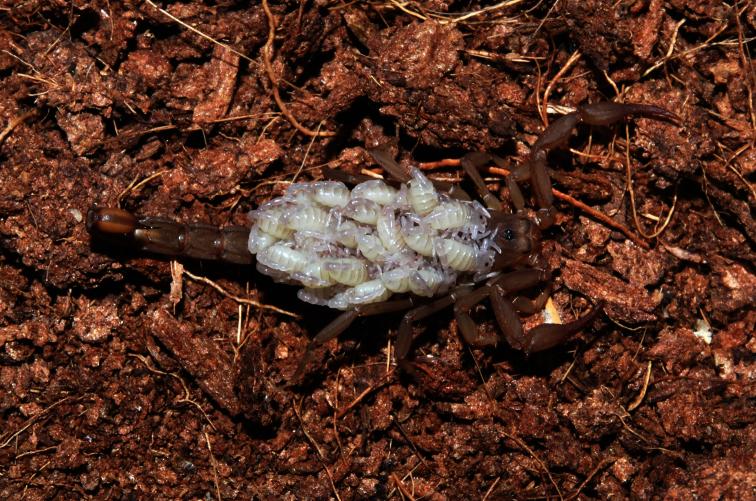
*Vaejovis brysoni*sp. n., paratype female with first instar juveniles.

**Figure 4. F4:**
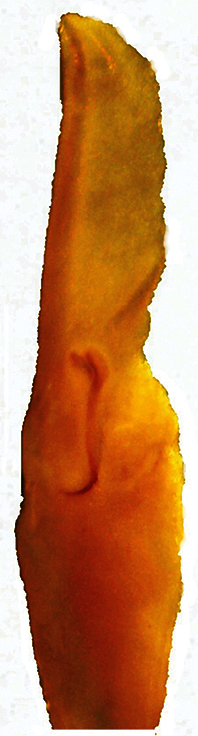
*Vaejovis brysoni*sp. n., paratype male right hemispermatophore.

**Figures 5–10.**
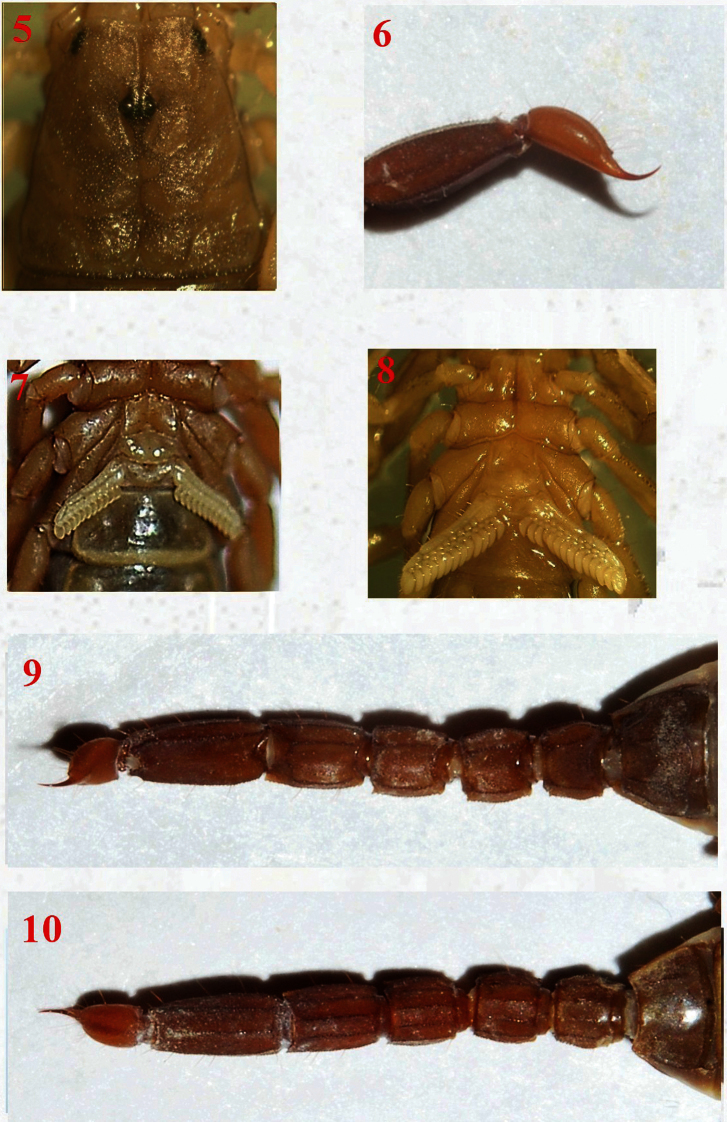
*Vaejovis brysoni*sp. n.,paratype female carapace **5**; telson **6**; pectines **7**; paratype male pectines and sternites **8**; paratype female metasoma dorsal **9**; and ventral **10**.

**Figure 11.**
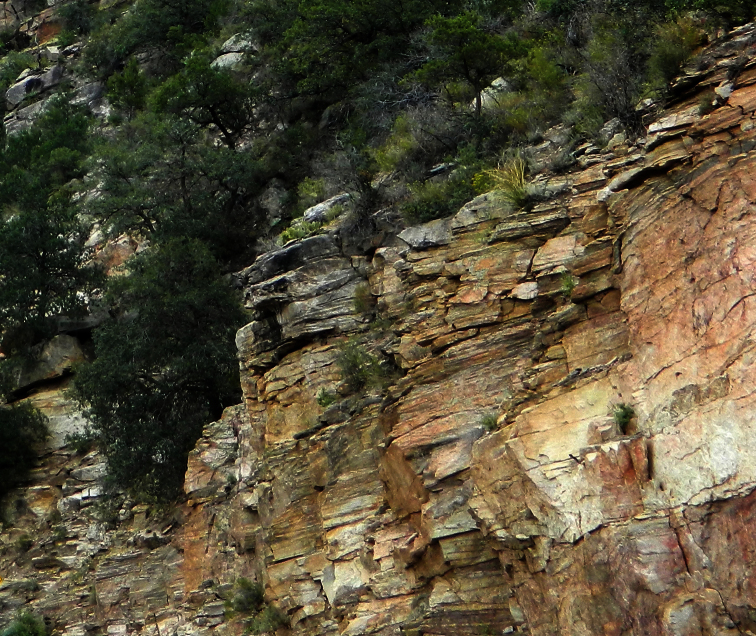
*Vaejovis brysoni*sp. n.habitat.

**Figure 12.**
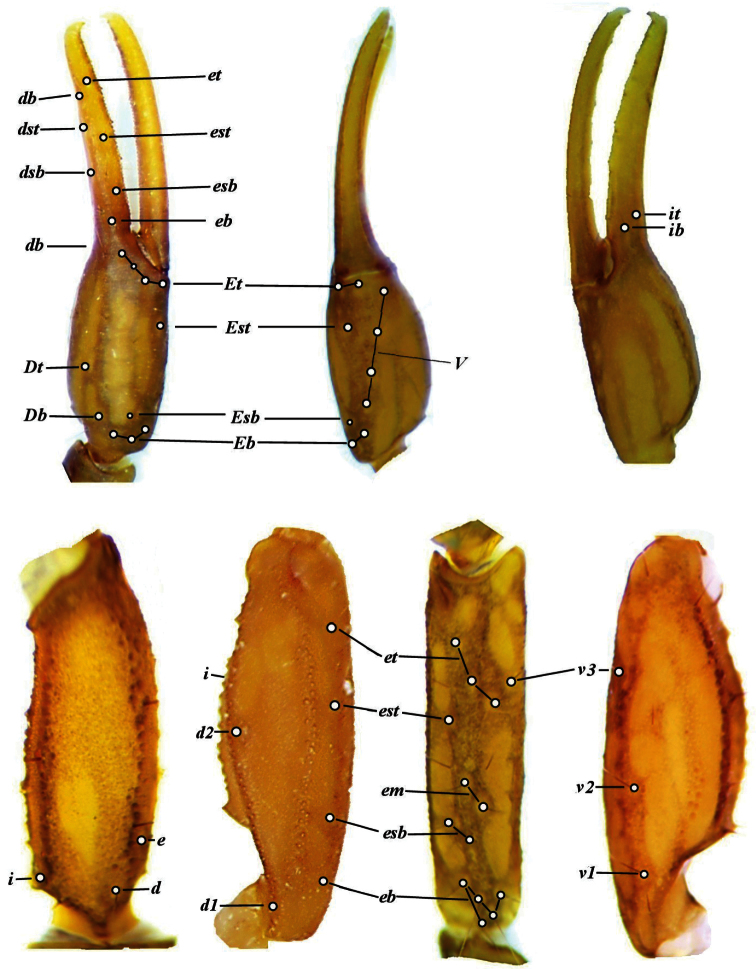
*Vaejovis brysoni*sp. n.trichobothrial pattern.

**Figure 13.**
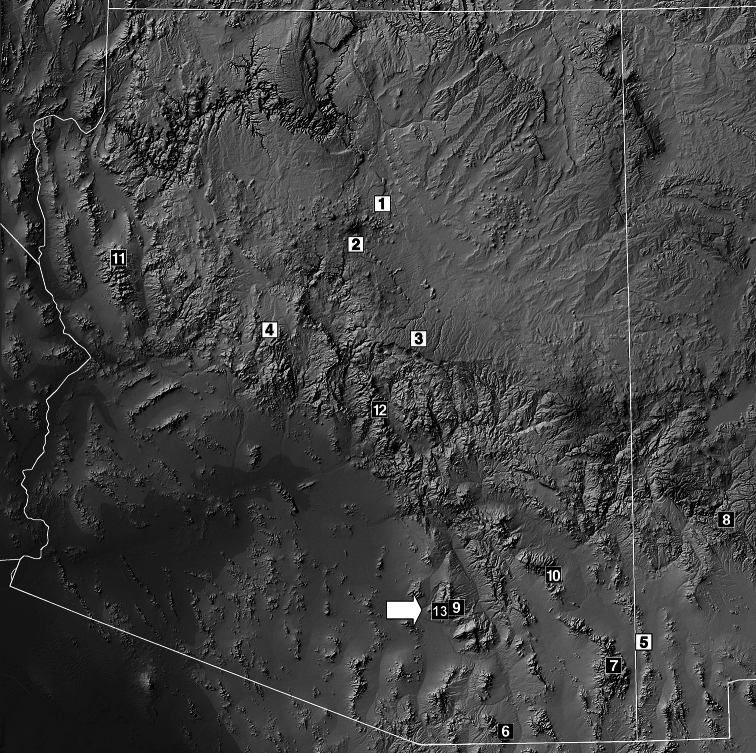
Map of Arizona and extreme western New Mexico showing the type localities of 13 of the 14 species in the *Vaejovis vorhiesi* group discussed in this paper, including the new species *Vaejovis brysoni* sp. n. The *Vaejovis bandido* type locality is south of Arizona in Sonora, Mexico. Localities are divided into those species exhibiting seven inner denticles (*ID*) on the chelal movable finger (white rectangles with black numbering) and those with primarily six *ID* denticles (black rectangles with white numbering). Seven *ID*s: **1** *Vaejovis jonesi* **2** *Vaejovis lapidicola* **3** *Vaejovis paysonensis* **4** *Vaejovis crumpi* and **5** *Vaejovis bigelowi*. Six *ID*s: **6** *Vaejovis vorhiesi* **7** *Vaejovis cashi* **8** *Vaejovis feti* **9** *Vaejovis deboerae* **10** *Vaejovis electrum* **11** *Vaejovis tenuipalpus* **12** *Vaejovis halli* and **13** *Vaejovis brysoni* sp. n.

## Supplementary Material

XML Treatment for Vaejovis brysoni
